# Synthesis and Herbicidal Activity of 5-(4-Hydroxybenzyl)-2-Thioxoimidazolidin-4-one Esters

**DOI:** 10.3390/molecules16042833

**Published:** 2011-03-30

**Authors:** Jintao Han, Jinmin Wang, Hongbo Dong, Jianping Lei, Mingan Wang, Jianxin Fang

**Affiliations:** 1 Department of Applied Chemistry, China Agricultural University, Beijing 100193, China; 2 State Key Laboratory of Elemento-Organic Chemistry, Nankai University, Tianjin 300071, China

**Keywords:** 5-(4-hydroxybenzyl)-2-thioxoimidazolidin-4-one esters, synthesis, herbicidal activity

## Abstract

A series of novel 5-(4-hydroxybenzyl)-2-thioxoimidazolidin-4-one esters were synthesized under mild conditions by the reaction of 5-(4-hydroxybenzyl)-2-thioxoimidazolidin-4-one and carboxylic acids with DCC and DMAP as the promoters. Their structures were confirmed by ^1^H-NMR, IR, ESI-MS and elemental analysis. The preliminary bioassy results indicated that some of compounds exhibit good herbicidal activity against *Zea mays*, *Triticum aestivum* and *Arabidopsis thaliana*. The further greenhouse test showed that compounds **6**-**16** and **6**-**28** have 60%, 50% and 50% efficacy against *Stellaria media, Echinochloa crus-galli and Setaria viridis* at 1,000 g/ha*,* respectively.

## 1. Introduction

Hydantoins and 2-thiohydantoin derivatives are an important class of biologically active molecules. They have not only been used in medicinal chemistry as anti-HSV [1], antidiabetic [2,3], or HDL-cholesterol modulators [4], but also used as fungicides [5] and herbicides [6,7] in agrochemical research. Among these hydantoin derivatives, hydantocidin (**1**) has generated great interest of chemists because of its excellent herbicide activity and low toxicity [8]. It is a non selective phytotoxin with an efficacy similar to that of glyphosate in contolling many common annual weeds [8]. 2-Thiohydantocidin (**2**) was also synthesized and found to have similar herbicidal activity [9]. Cseke and Waters [10,11] found that hydantocidin acts as a proherbicide, which is phosphorylated at the 5’ position *in vivo*. In the phosphorylated form **3**, it strongly binds to its target enzyme, adenylosuccinate synthetase (AdSS, EC 6.3.3.4) [12,13,14], a new herbicidal target. This finding led to a great of attention being paid toward this compound or its analogues as potential lead compounds in the discovery of new commercial herbicides [15,16,17,18]. In the investigation of 5’-phosphohydantocidin analogues as AdSS inhibitors, Crouse found that **4** had significant inhibition against *Arabidopsis*, with an I_50_ value of 0.005 mg/L, and inhibition against eight grass and broadleaf weed species with post GR_50_ values of 16.6 mg/L, which are much higher than those of 0.5 mg/L and 29 mg/L obtained for hydantocidin [19]. In our laboratory, a virtual screen model was set up based on the docking method of inhibitors with AdSS, and compared with the crystal structure of the AdSS-hydantocidin complex [14,20]. As a continuation of our ongoing project aimed at looking for novel biologically imidazolinedione heterocyclic compounds [21,22,23], a series of novel 5-(4-hydroxybenzyl)-2-thioxoimidazolidin-4-one esters were synthesized, and their activities were evaluated. Herein, we would like to report the synthesis and biological activity of the title compounds.

**Scheme 1:**
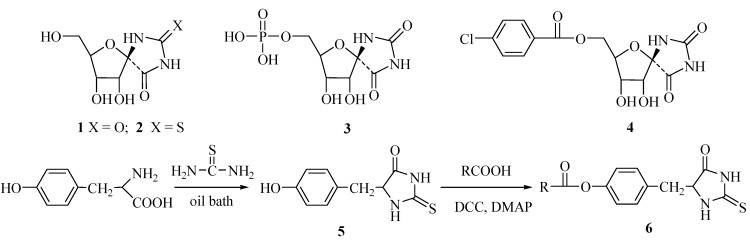
The synthetic route to compounds **6**.

## 2. Results and Discussion

**Figure 1:**
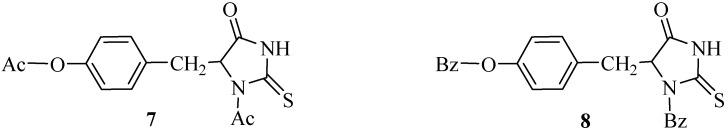
Reaction byproducts **7** and **8**.

5-(4-Hydroxybenzyl)-2-thioxoimidazolidin-4-one (**5**) was prepared by heating L-tyrosine and thiourea in an oil bath under reflux according to the reported method [24]. Compound **5** reacted at room temperature with various substituted benzoic acids with DCC and DMAP as catalysts to give the target compounds **6** in yields of 27-74%. Their structures were confirmed by IR, ^1^H-NMR, ESI-MS and elemental analysis. As reported before [21], when acetic anhydride or benzoyl chloride were used, compounds **7** and **8** (Figure 1) were produced as byproducts.

The herbicidal values of the title compounds against *Zea mays*, *Triticum aestivum* and *Arabidopsis thaliana* at the dosages of 200 μg/mL were assayed, and compared with the commercially available herbicide metazachlor as positive control according to the method described in the Experimental section. The activity data are listed in Table 1. 

**Table 1:** The inhibition ratios (%) of **6** against *Z.* *mays*, *T. aestivum* and *A. thaliana* at 200 μg/mL.

**Compd.**	R	*Z. mays*		*T. aestivum*		*A. thaliana*
		Height	Root	Height	Root	Germination
CK		-	-	-	-	0
metazachlor		79.0	83.0	78.6	77.7	70
**6-1**	CH_3_-	-14.5	-6.7	-3.6	20.9	10
**6-2**	4-FC_6_H_4_-	32.8	66.3	-22.9	-8.5	20
**6-3**	C_6_H_5_-	26.6	44.5	-11.1	16.2	10
**6-4**	3-NO_2_C_6_H_4_-	3.9	-16.3	20.6	16.3	20
**6-5**	2-FC_6_H_4_-	-19.4	-30.2	48.4	57.9	30
**6-6**	2,4-(OCH_3_)_2_C_6_H_3_-	19.6	-11.6	-2.9	10.7	20
**6-7**	2,3-(OCH_3_)_2_C_6_H_3_-	6.7	11.2	-10.9	20.9	20
**6-8**	2-ClC_6_H_4_-	53.4	5.9	24.7	53.4	30
**6-9**	2-NO_2_C_6_H_4_-	63.6	7.6	3.7	10.6	40
**6-10**	3-ClC_6_H_4_-	41.7	22.1	8.8	47.5	70
**6-11**	4-BrC_6_H_4_-	14.7	22.0	5.2	-48	60
**6-12**	4-ClC_6_H_4_-	23.5	8.6	-12.9	-13.6	40
**6-13**	3,4-Cl_2_C_6_H_3_-	36.7	24.1	-15. 8	7.0	40
**6-14**	4-Cl-3-NO_2_C_6_H_3_-	54.4	44.2	-3.7	2.6	50
**6-15**	3-CH_3_C_6_H_4_-	62.8	61.3	-14.8	12.7	50
**6-16**	4-NO_2_C_6_H_4_-	100	100	-24.6	-25.8	40
**6-17**	4-OCH_3_C_6_H_4_-	33.5	17.8	25.1	32.2	30
**6-18**	4-CH_3_C_6_H_4_-	27.9	38.7	1.27	16.8	60
**6-19**	2-CH_3_C_6_H_4_-	53.2	33.5	5.8	20.7	60
**6-20**	3,4-(OCH_3_)_2_C_6_H_3_-	57.9	64.2	26.1	43.1	50
**6-21**	C_6_H_5_CH_2_-	53.9	58.1	34.9	42.1	20
**6-22**	2,4-Cl_2_C_6_H_3_OCH_2_-	65.5	74.9	97.8	99.6	50
**6-23**	2-OCH_3_C_6_H_4_-	-26.2	-29.0	81.3	79.1	50
**6-24**	4-ClC_6_H_4_OCH_2_-	16.4	59.4	95.2	99.6	40
**6-25**	3,4,5-(OCH_3_)_3_C_6_H_2_-	22.1	19.9	42.3	59.7	10
**6-26**	4-C_6_H_5_C_6_H_4_-	42.4	48.7	20.9	3.2	5
**6-27**	4- *t*-C_4_H_9_C_6_H_4_-	30.9	22.5	55.5	25.9	10
**6-28**	1-C_10_H_7_CH_2_-	77.4	88.0	100	56.7	10
**6-29**	3,5-(NO_2_)_2_C_6_H_3_-	27.8	71.0	86.2	18.8	5
**6-30**	2-C_4_H_3_S-	11.6	-24.7	-1.2	15.5	20
**6-31**	C_6_H_5_CH=CH-	52.7	77.9	33.8	2.5	10
**6-32**	3,5-(CF_3_)_2_C_6_H_3_CH_2_-	45.5	71.5	65.5	32.7	5
**6-33**	4-FC_6_H_4_CH_2_-	70.1	77.3	50.2	86.6	20
**6-34**	2-C_4_H_3_O-	72.8	60.7	27.5	30.3	40

The preliminary bioassay results showed that some of the compounds **6** possess good herbicidal activity. For example, compounds **6**-**16**, **6**-**28**, **6**-**33** and **6**-**34** exhibited more than 70% inhibitory activity against the height growth of *Z. mays*, and compounds **6**-**16**, **6**-**22**, **6**-**28**, **6**-**29**, **6**-**31**, **6**-**32** and **6**-**33** exhibited more than 70% inhibitory activity against the root growth of *Z. mays*, while compounds **6**-**22**, **6**-**23**, **6**-**24**, **6**-**28** and **6**-**29** showed more than 75% inhibitory activity against the height growth of *T. aestivum*, and compounds **6**-**22**, **6**-**23**, **6**-**24** and **6**-**33** showed more than 75% inhibitory activity against the root growth of *T. aestivum*. These values are almost equal to or better than those of metazachlor, the commercially available herbicide. Also the inhibitory ratios of **6**-**10**, **6**-**11**, **6**-**18** and **6**-**19** are equal or close to those of metazachlor against the germination of *A. thaliana*, the *A. thaliana* leaves exhibited yellowing and withering symptoms after germination. These results indicated that these compounds have selectivity as potential herbicide. Compared with the results in the previous paper [21,22], the inhibitory activities of **6** against *Z. mays*, *T. aestivum* and *A. thaliana* were increased, the growth promotion activities were inhibited, which showed the thiohydantoin ring is more helpful to increase the herbicidal activity than hydantoin ring. Comparison the activities of **7** and **8** with those of **6**-**1** and **6**-**3**, they are greatly decreased due to the loss one of the N-H groups in the thiohydantoin ring, which is consistent with the results in the previous paper [21,22] and the X-ray structure of the AdSS-hydantocidin complex [14]. Further greenhouse tests showed that **6**-**16** and **6**-**28** have 60%, 50% and 50% efficacy against *Stellaria media, Echinochloa crus-galli* and *Setaria viridis* at the dosage of 1,000 g/ha when used as a pre-emergence treatment, but no significant efficacy was observed with 0%, 0% and 0% efficacy against *S. media, E. crus-galli* and *S. viridis* when used as a post-emergence treatment. Further structure modifications are under investigation.

## 3. Experimental

### 3.1. General

Meting points were measured on a Yanagimoto apparatus (Yanagimoto MFG CO., Japan) and are uncorrected. Elemental analysis was performed on a Vario EL instrument (Elementar Vario Micro Cube, Germany). The ^1^H NMR spectra were recorded at room temperature on a Bruker DPX 300 spectrometer with DMSO-*d*_6_ as a solvent and TMS as an internal standard. IR spectra were obtained on a Shimadzu IR-435 instrument with KBr plates. ESI-MS were analyzed on an Agilent 1100 LC-MSD-Trap instrument. Herbicidal activity tests were carried out in a RXZ-380B illumination incubator (Jiangnan Equipment Factory, Ningbo, Zhejiang, China).

### 3.2. Synthesis

#### 3.2.1. Synthesis of 5-[(4-hydroxyphenyl)methyl]-2-thioxoimidazolidin-4-one (**5**)

A mixture of L-tyrosine (5.4 g, 30 mmol) and thiourea (6.9 g, 90 mmol) was placed in a flask and heated under stirring with the oil bath. The oil bath temperature was controlled at 180-190 °C, and about 5 min later the homogenous liquid started to fume. After 15 minutes, the reaction was complete as monitored by TLC. The flask was allowed to cool down and water (20 mL) was added when the flask was still warm. The solution was reheated to dissolve all the solids and allowed to cool to room temperature, then placed in a refrigerator for 4 h. The crystals of compound **5** were removed by vacuum filtration, and the mother liquid was extracted with ethyl acetate and further purified by flash column chromatography. Finally, 5.3 g product was obtained (80% yield), m.p.208-210 °C (211 °C, lit [24]). ^1^H-NMR (DMSO-*d*_6_, 300 MHz ) *δ*: 11.39 (s, 1H, NH), 10.01 (s, 1H, NH), 9.25 (s, 1H, OH), 6.94 (d, *J* = 8.7 Hz, 2H, Ar-H), 6.63 (d, *J* = 8.7 Hz, 2H, Ar-H), 4.45 (dd, *J* = 3.9, 4.8 Hz, 1H, CH), 2.85-2.87 (m, 2H, CH_2_). IR: 3440, 3289, 1752, 1744, 1615, 1595, 1441, 1260, 1061 cm^-1^.

#### 3.2.2. General Procedure for the Synthesis of Compounds **6**

To a stirred solution of 5-[(4-hydroxy phenyl)methyl]-2-thioxoimidazolidin-4-one (**5**, 2 mmol) and a substituted benzoic acid (2.4 mmol) in dry actone (25 mL), DCC (0.49 g, 2.4 mmol) and DMAP (0.02 g, 0.2 mmol) were added at room temperature. After about 8 h, the reaction is almost complete as monitored by TLC. After filtration and concentration under vacuum, the crude product was purified by column chromatography on silica gel (petroleum ether-EtOAc 5:1-2:1) or recrystallized from ethanol to give yellow solid compounds **6**.

*5-[[4-(Acetyloxy)phenyl]methyl]-2-thioxo-4-imidazolidinone* (**6**-**1**). Yield 65%, mp: 209-211 °C. ^1^H- NMR (DMSO-*d*_6_) *δ*: 2.24 (s, 3H, CH_3_), 2.97 (m, 2H, CH_2_), 4.55 (dd, J = 4.2, 5.1 Hz, 1H, CH), 7.04 (d, J = 8.7 Hz, 2H, Ar-H), 7.19 (d, J = 8.7 Hz, 2H, Ar-H), 10.09 (s, 1H, NH), 11.50 (s, 1H, NH); IR: 3450, 3172, 1745, 1732, 1608, 1555, 1363, 1289, 1008 cm^-1^. Anal calcd. for C_12_H_12_N_2_O_3_S: C, 54.53, H, 4.58, N, 10.60; found: C, 54.65, H, 4.54, N, 10.57.

*5-[[4-(4-Fluorobenzoyloxy)phenyl]methyl]-2-thioxo-4-imidazolidinone* (**6**-**2**). Yield 59%, mp: 197-198 °C. ^1^H-NMR (DMSO-*d*_6_): δ 3.02 (m, 2H, CH_2_), 4.59 (dd, J = 4.2, 5.0 Hz, 1H, CH), 7.27-7.19 (m, 4H, Ar-H), 7.41-8.22 (m, 4H, Ar-H), 10.12 (s, 1H, NH), 11.52 (s, 1H, NH); IR: 3448, 3163, 1752, 1739, 1605, 1550, 1386, 1265, 1070 cm^-1^. Anal calcd. for C_17_H_13_FN_2_O_3_S: C, 59.29, H,3.81, N, 8.13; found: C, 59.23, H, 3. 88, N, 8.18.

*5-[4-(Benzoyloxy)phenyl]methyl]-2-thioxo-4-imidazolidinone* (**6**-**3**). Yield 60%, mp: 198-199 °C. ^1^H- NMR (DMSO-*d*_6_): δ 3.02 (m, 2H, CH_2_), 4.59 (dd, J = 4.2, 5.1 Hz, 1H, CH), 7.27-7.19 (m, 4H, Ar-H), 7.60-8.14 (m, 5H, Ar-H), 10.12 (s, 1H, NH), 11.52 (s, 1H, NH); IR: 3467, 3255, 1763, 1739, 1606,1588, 1446, 1260, 1015 cm^-1^. Anal calcd. for C_17_H_14_N_2_O_3_S: C, 62.56, H, 4.32, N, 8.58; found: C, 62.33, H, 4.38, N, 8.39.

*5-[[4-(3-Nitrobenzoyloxy)phenyl]methyl]-2-thioxo-4-imidazolidinone* (**6**-**4**). Yield 59%, mp: 210-211 °C. ^1^H- NMR (DMSO-*d*_6_): δ3.01-3.03 (m, 2H, CH_2_), 4.60 (dd, J = 4.2, 5.0 Hz, 1H, CH), 7.25-7.31 (m, 4H, Ar-H), 7.89-7.94 (m, 3H, Ar-H), 8.52-8.60 (m, 2H, Ar-H), 8.77-8.79 (m, 1H, Ar-H), 10.13 (s, 1H, NH), 11.53 (s, 1H, NH); IR: 3446, 3154, 1702, 1729, 1614, 1541, 1350, 1250, 1005 cm^-1^. Anal calcd. for C_17_H_13_FN_3_O_5_S: C, 54.98, H, 3.53, N, 11.31; found: C, 55.02, H, 3. 73, N, 11.08.

*5-[[4-(2-Fluorobenzoyloxy)phenyl]methyl]-2-thioxo-4-imidazolidinone* (**6-5**). Yield 30%, mp: 199-200 °C. ^1^H-NMR (DMSO-*d*_6_): δ 3.01-3.03 (m, 2H, CH_2_), 4.59 (dd, J = 4.2, 5.1 Hz, 1H, CH), 7.20-7.28 (m, 4H, Ar-H), 7.38-7.47 (m, 2H, Ar-H), 7.74-7.81 (m, 1H, Ar-H), 8.06-8.12 (m, 1H, Ar-H), 10.12 (s, 1H, NH), 11.52 (s, 1H, NH); IR: 3447, 3162, 1737, 1728, 1613, 1547, 1454, 1298, 1065 cm^-1^. Anal calcd. for C_17_H_13_FN_2_O_3_S: C, 59.29, H, 3.81, N, 8.13; found: C, 59.23, H, 3. 88, N, 8.18.

*5-[[4-(2,4-Dimethoxybenzoyloxy)phenyl]methyl]-2-thioxo-4-imidazolidinone* (**6**-**6**). Yield 27%, mp: 169-171 °C. ^1^H-NMR (DMSO-*d*_6_): δ 3.00-3.03 (m, 2H, CH_2_), 3.85 (s, 3H, OCH_3_), 3.86 (s, 3H, OCH_3_), 4.58 (dd, *J* = 5.1, 4.2 Hz, 1H, CH), 6.64-6.70 (m, 2H, Ar-H), 7.11 (d, *J* = 8.4 Hz, 2H, Ar-H), 7.23 (d, *J* = 8.4 Hz, 2H, Ar-H), 7.93 (d, *J* = 8.7 Hz, 1H, Ar-H), 10.11 (s, 1H, NH), 11.50 (s, 1H, NH); IR: 3459, 3138, 1739, 1732, 1605, 1555, 1405, 1246, 1010 cm^-1^. Anal calcd. for C_19_H_18_N_2_O_5_S: C, 59.06, H, 4.70, N, 7.25; found: C, 59.07, H, 4.75, N, 7.15.

*5-[[4-(2,3-Dimethoxybenzoyloxy)phenyl]methyl]-2-thioxo-4-imidazolidinone* (**6**-**7**). Yield 34%, mp: 191-193 °C. ^1^H-NMR (DMSO-*d*_6_): δ 3.01-3.03(m, 2H, CH_2_), 3.82 (s, 3H, OCH_3_), 3.87 (s, 3H, OCH_3_ ), 4.58-4.60 (m, 1H, CH), 7.17-7.27 (m, 5H, Ar-H), 7.33-7.42 (m, 2H, Ar-H), 10.12 (s, 1H, NH), 11.52 (s, 1H, NH); IR: 3450, 3173, 1737, 1728, 1586, 1525, 1384, 1274, 1043 cm^-1^. Anal calcd. for C_19_H_18_N_2_O_5_S: C, 59.06, H, 4.70, N, 7.25; found: C, 58.82, H, 4.77, N, 7.32.

*5-[[4-(2-Chlorobenzoyloxy)phenyl]methyl]-2-thioxo-4-imidazolidinone* (**6**-**8**). Yield 47%, mp: 196-198 °C. ^1^H-NMR (DMSO-*d*_6_): δ 3.01-3.04 (m, 2H, CH_2_), 4.59 (dd, *J* = 4.2, 5.1 Hz, 1H, CH), 7.21-7.29 (m, 4H, Ar-H), 7.52-7.57 (m , 1H, Ar-H), 7.65-7.68 (m, 2H, Ar-H), 8.05-8.09 (m, 1H, Ar-H), 10.12 (s, 1H, NH), 11.50 (s, 1H, NH); IR: 3447, 3178, 1741, 1730, 1590, 1439, 1398, 1293, 1038 cm^-1^. Anal calcd. for C_17_H_13_ClN_2_O_3_S: C, 56.59, H, 3.63, N, 7.76; found: C, 56.68, H, 3.73, N, 7.68.

*5-[[4-(2-Nitrobenzoyloxy)phenyl]methyl]-2-thioxo-4-imidazolidinone* (**6**-**9**). Yield 51%, mp: 213-214 °C. ^1^H- NMR (DMSO-*d*_6_): δ 3.01-3.03 (m, 2H, CH_2_), 4.58 (dd, *J* = 4.2, 5.1 Hz, 1H, CH), 7.19 (d, *J* = 8.4 Hz, 2H, Ar-H), 7.30 (d, *J* = 8.4 Hz, 2H, Ar-H), 7.88-7.98 (m, 2H, Ar-H), 8.07-8.11 (m, 1H, Ar-H), 8.17-8.20 (m, 1H, Ar-H), 10.12 (s, 1H, NH), 11.52 (s, 1H, NH); IR: 3345, 3169, 1741, 1728, 1610, 1537, 1398, 1294, 1060 cm^-1^. Anal calcd. for C_17_H_13_N_3_O_5_S: C, 54.98, H, 3.53, N, 11.31; found: C, 54.22, H, 3.57, N, 12.31.

*5-[[4-(3-Chlorobenzoyloxy)phenyl]methyl]-2-thioxo-4-imidazolidinone* (**6**-**10**). Yield 42%, mp: 192-193 °C. ^1^H-NMR (DMSO-*d*_6_): δ 3.02-3.04 (m, 2H, CH_2_), 4.59 (dd, *J* = 4.2, 5.1 Hz, 1H, CH), 7.21-7.28 (m, 4H, Ar-H), 7.62-7.67 (m , 1H, Ar-H), 7.81-7.85 (m, 1H, Ar-H), 8.06-8.11 (m, 2H, Ar-H), 10.12 (s, 1H, NH), 11.51 (s, 1H, NH); IR: 3343, 3155, 1741, 1735, 1625, 1546, 1408, 1289, 1017 cm^-1^. Anal calcd. for C_17_H_13_ClN_2_O_3_S: C, 56.59, H, 3.63, N, 7.76; found: C, 56.62, H, 3.72, N, 7.70.

*5-[[4-(4-Bromobenzoyloxy)phenyl]methyl]-2-thioxo-4-imidazolidinone* (**6**-**11**). Yield 47%, mp: 242-244 °C. ^1^H-NMR (DMSO-*d*_6_): δ 3.01-3.03 (m, 2H, CH_2_), 4.59 (dd, *J* = 4.2, 5.1 Hz, 1H, CH), 7.20-7.28 (m, 4H, Ar-H), 7.82 (m, 2H, Ar-H), 8.03-8.06 (m, 2H, Ar-H), 10.13 (s, 1H, NH), 11.52 (s, 1H, NH); IR: 3288, 3142, 1770, 1741, 1604, 1515, 1382, 1266, 1066 cm^-1^. Anal calcd. for C_17_H_13_BrN_2_O_3_S: C, 50.38, H, 3.23, N, 6.91; found: C, 50.31, H, 3.55, N, 6.99. ESI-MS m/z: 405, 407 [M+H]^+^.

*5-[[4-(4-Chlorobenzoyloxy)phenyl]methyl]-2-thioxo-4-imidazolidinone* (**6**-**12**). Yield 42%, mp: 226-228 °C. ^1^H-NMR (DMSO-*d*_6_): δ 3.01-3.03 (m, 2H, CH_2_), 4.59 (dd, *J* = 4.2, 5.1 Hz, 1H, CH), 7.20-7.28 (m, 4H, Ar-H), 7.67-7.71 (m, 2H, Ar-H), 8.11-8.15 (m, 2H, Ar-H), 10.14 (s, 1H, NH), 11.53 (s, 1H, NH); IR: 3152, 3167, 1738, 1729, 1600, 1592, 1400, 1263, 1077 cm^-1^. Anal calcd. for C_17_H_13_ClN_2_O_3_S: C, 56.59, H, 3.63, N, 7.76; found: C, 56.86, H, 3.86, N, 7.73.

*5-[[4-(3,4-Dicholorobenzoyloxy)phenyl]methyl]-2-thioxo-4-imidazolidinone* (**6**-**13**). Yield 53%, mp: 200-202 °C. ^1^H-NMR (DMSO-*d*_6_): δ 3.02-3.04 (m, 2H, CH_2_), 4.58 (dd, *J* = 4.2, 5.1 Hz, 1H, CH), 7.22-7.29 (m, 4H, Ar-H), 7.64-7.68 (dd, *J* = 2.1, 8.6 Hz, 1H, Ar-H), 7.88 (d, *J* = 2.1 Hz, 1H, Ar-H), 8.12 (d, *J* = 8.6 Hz, 2H, Ar-H), 10.13 (s, 1H, NH), 11.52 (s, 1H, NH); IR: 3348, 3150, 1752, 1741, 1584, 1546, 1400, 1240, 1096 cm^-1^. Anal calcd. for C_17_H_12_Cl_2_N_2_O_3_S: C, 51.66, H, 3.06, N, 7.09; found: C, 52.25, H, 3.42, N, 7.26.

5-[[4-(4-Chloro-3-nitro-benzoyloxy)phenyl]methyl]-2-thioxo-4-imidazolidinone (**6**-**14**). Yield 41%, mp: 230-232 °C. ^1^H-NMR (DMSO-*d*_6_): δ 2.98-3.04 (m, 2H, CH_2_), 4.58 (dd, *J* = 4.2, 5.1 Hz, 1H, CH), 7.18-7.30 (m, 4H, Ar-H), 8.02 (d, *J* = 8.5 Hz, 1H, Ar-H), 8.36 (dd, *J* = 2.0, 8.5 Hz, 1H, Ar-H), 8.72 (d, *J* = 2.0 Hz, 1H, Ar-H), 10.13 (s, 1H, NH), 11.52 (s, 1H, NH); IR: 3443, 3154, 1762, 1735, 1602, 1541, 1402, 1240, 1093 cm^-1^. Anal calcd. for C_17_H_12_ClN_3_O_5_S: C, 50.31, H, 2.98, N, 10.35; found: C, 50.62, H, 3.20, N, 10.34.

*5-[[4-(3-Methylbenzoyloxy)phenyl]methyl]-2-thioxo-4-imidazolidinone* (**6**-**15**). Yield 51%, mp: 163-165 °C. ^1^H-NMR (DMSO-*d*_6_): δ 2.42 (s, 1H, CH_3_), 3.02-3.04 (m, 2H, CH_2_), 4.59 (dd, *J* = 4.2, 5.0 Hz, 1H, CH), 7.27-7.17 (m, 4H, Ar-H), 7.46-7.58 (m, 2H, Ar-H), 7.91-7.95 (m, 2H, Ar-H), 10.13 (s, 1H, NH), 11.53 (s, 1H, NH); IR: 3459, 3188, 1754, 1743, 1604, 1545, 1382, 1266, 1026 cm^-1^. Anal calcd. for C_18_H_16_N_2_O_3_S: C, 63.51, H, 4.74, N, 8.23; found: C, 63.24, H, 4. 92, N, 8.32.

*5-[[4-(4-Nitrobenzoyloxy)phenyl]methyl]-2-thioxo-4-imidazolidinone* (**6**-**16**). Yield 42%, mp: 246-248 °C. ^1^H-NMR (DMSO-*d*_6_): δ 3.02-3.04 (m, 2H, CH_2_), 4.59 (dd, *J* = 4.2, 5.1 Hz, 1H, CH), 7.25-7.28 (m, 4H, Ar-H), 8.35-8.44 (m, 4H, Ar-H), 10.13 (s, 1H, NH), 11.53 (s, 1H, NH); IR: 3281, 3158, 1759, 1732, 1603, 1523, 1395, 1269, 1082 cm^-1^. Anal calcd. for C_17_H_13_N_3_O_5_S: C, 54.98, H, 3.53, N, 11.31; found: C, 55.13, H, 3.82, N, 11.03.

*5-[[4-(4-Methoxybenzoyloxy)pheny]*methyl]-2-thioxo-4-imidazolidinone** (**6**-**17**). Yield 61%, mp: 226-228 °C. ^1^H NMR (DMSO-*d*_6_): δ 3.01-3.03 (m, 2H, CH_2_), 3.87 (s, 3H, OCH_3_), 4.58 (dd, *J* = 4.2, 5.1 Hz, 1H, CH), 7.10-7.19 (m, 4H, Ar-H), 7.23-7.26 (m, 2H, Ar-H), 8.05-8.10 (m, 2H, Ar-H), 10.12 (s, 1H, NH), 11.51 (s, 1H, NH); IR: 3281, 3158, 1759, 1732, 1603, 1523, 1395, 1269, 1070 cm^-1^. Anal calcd. for C_17_H_13_N_3_O_5_S: C, 54.98, H, 3.53, N, 11.31; found: C, 55.13, H, 3.82, N, 11.03. ESI-MS m/z: 357 [M+H]^+^, 379 [M+Na]^+^.

*5-[[4-(4-Methylbenzoyloxy)phenyl]methyl]-2-thioxo-4-imidazolidinone* (**6**-**18**). Yield 52%, mp: 234-236 °C. ^1^H-NMR (DMSO-*d*_6_): δ 2.43 (s, 3H, CH_3_), 3.02-3.04 (m, 2H, CH_2_), 4.59 (dd, *J* = 4.2, 5.1 Hz, 1H, CH), 7.17-7.27 (m, 4H, Ar-H), 7.40 (d, *J* = 8.5 Hz, 2H, Ar-H), 8.01 (d, *J* = 8.5 Hz, 2H, Ar-H), 10.12 (s, 1H, NH), 11.51 (s, 1H, NH); IR: 3350, 3188, 1762, 1740, 1604, 1515, 1382, 1266, 1066 cm^-1^. Anal calcd. for C_18_H_16_N_2_O_3_S: C, 63.51, H, 4.74, N, 8.23; found: C, 63.63, H, 4.84, N, 8.17. ESI-MS m/z: 341 [M+H]^+^, 363 [M+Na]^+^.

*5-[[4-(2-Methylbenzoyloxy)phenylmethyl]*-2-thioxo-4-imidazolidinone** (**6**-**19**). Yield 35%, mp: 190-192 °C. ^1^H-NMR (DMSO-*d*_6_): δ 2.59 (s, 1H, CH_3_), 3.01-3.03 (m, 2H, CH_2_), 4.59 (dd, *J* = 4.2, 5.1 Hz, 1H, CH), 7.17-7.28 (m, 4H, Ar-H), 7.37-7.42 (m, 2H, Ar-H), 7.55-7.60 (m, 1H, Ar-H), 8.04-8.08 (m, 1H, Ar-H), 10.12 (s, 1H, NH), 11.52 (s, 1H, NH); IR: 3348, 3162, 1760, 1737, 1601, 1545, 1398, 1266, 1046 cm^-1^. Anal calcd. for C_18_H_16_N_2_O_3_S: C, 63.51, H, 4.74, N, 8.23; found: C, 63.53, H, 4.79, N, 8.21.

*5-[[4-(3,4-Dimethoxybenzoyloxy)phenyl]methyl]-2-thioxo-4-imidazolidinone* (**6**-**20**). Yield 44%, mp: 228-230 °C. ^1^H-NMR (DMSO-*d*_6_): δ 3.01-3.03 (m, 2H, CH_2_), 3.84 (s, 3H, OCH_3_), 3.87 (s, 3H, OCH_3_), 4.59 (dd, *J* = 4.2, 5.1 Hz, 1H, CH), 7.14-7.26 (m, 5H, Ar-H), 7.58 (d, *J* = 2.0 Hz, 1H, Ar-H), 7.76-7.79 (m, 1H, Ar-H), 10.12 (s, 1H, NH), 11.52 (s, 1H, NH); IR: 3498, 3186, 1758, 1749, 1596, 1520, 1409, 1279, 1017 cm^-1^. Anal calcd. for C_19_H_18_N_2_O_5_S: C, 59.06, H, 4.70, N, 7.25; found: C, 58.94, H, 4.62, N, 7.21. ESI-MS m/z: 385 [M-H]^-^.

*5-[[4-(Phenylacetyloxy)phenyl]methyl]-2-thioxo-4-imidazolidinone* (**6**-**21**). Yield 39%, mp: 160-162 °C. ^1^H- NMR (DMSO-*d*_6_): δ 2.97-2.99 (m, 2H, CH_2_), 3.94 (s, 2H, CH_2_Ar), 4.58 (dd, *J* = 4.2, 5.1 Hz, 1H, CH), 7.03-7.19 (m, 4H, Ar-H), 7.28-7.37 (m, 5H, Ar-H), 10.12 (s, 1H, NH), 11.52 (s, 1H, NH); IR: 3378, 3159, 1756, 1748, 1548, 1507, 1320, 1199, 1010 cm^-1^. Anal calcd. for C_18_H_16_N_2_O_3_S: C, 63.51, H, 4.74, N, 8.23; found: C, 63.45, H, 4.77, N, 8.19. ESI-MS m/z: 339 [M-H]^-^.

*5-[[4-(2,4-Dichlorophenoxyacetyl)phenyl]methyl]-2-thioxo-4-imidazolidinone* (**6**-**22**). Yield 60%, mp: 150-152 °C. ^1^H-NMR (DMSO-*d*_6_): δ 2.98-3.01 (m, 2H, CH_2_), 4.58 (dd, *J* = 4.2, 5.1 Hz, 1H, CH), 5.23 (s, 2H, OCH_2_), 7.10-7.29 (m, 5H, Ar-H), 7.38-7.63 (m, 2H, Ar-H), 10.11 (s, 1H, NH), 11.51 (s, 1H, NH); IR: 3450, 3150, 1752, 1743, 1552, 1489, 1308, 1201, 1089 cm^-1^. Anal calcd. for C_18_H_14_Cl_2_N_2_O_4_S: C, 50. 83, H, 3.32, N, 6.59; found: C, 50.86, H, 3.51, N, 6.75. ESI-MS m/z: 425, 423 [M-H]^-^.

*5-[[4-(2-Methoxybenzoyloxy)phenyl]methyl]-2-thioxo-4-imidazolidinone* (**6**-**23**). Yield 58%, mp: 158-160 °C. ^1^H-NMR (DMSO-*d*_6_): δ 2.86-2.88 (m, 2H, CH_2_), 3.87 (s, 3H, OCH_3_), 4.57 (dd, *J* = 4.2, 5.0 Hz, 1H, CH), 6.96-7.17 (m, 3H, Ar-H), 7.21-7.26 (m, 3H, Ar-H), 7.60-7.66 (m, 1H, Ar-H), 7.87-7.90 (m, 1H, Ar-H), 10.10 (s, 1H, NH), 11.51 (s, 1H, NH); IR: 3470, 3173, 1842, 1760, 1593, 1487, 1348, 1253, 1047 cm^-1^. Anal calcd. for C_18_H_16_N_2_O_4_S: C, 60. 66, H, 4.53, N, 7.86; found: C, 60.39, H, 4.57, N, 7.95.

*5-[[4-(4-Chlorophenoxyacetyl)phenyl]methyl]-2-thioxo-4-imidazolidinone* (**6**-**24**). Yield 53%, mp: 184-186 °C. ^1^H-NMR (DMSO-*d*_6_): δ 2.99-3.01 (m, 2H, CH_2_), 4.57 (dd, *J* = 4.2, 5.1 Hz, 1H, CH), 5.08 (s, 2H, OCH_2_), 7.04-7.11 (m, 4H, Ar-H), 7.23 (d, *J* = 8.6 Hz, 2H, Ar-H), 7.34-7.38 (d, *J* = 8.6 Hz, 2H, Ar-H), 10.11 (s, 1H, NH), 11.51 (s, 1H, NH); IR: 3435, 3149, 1850, 1737, 1547, 1495, 1320, 1170, 1072 cm^-1^. Anal calcd. for C_18_H_15_ClN_2_O_4_S: C, 55. 31, H, 3.87, N, 7.17; found: C, 55.48, H, 4.16, N, 7.29.

*5-[[4-(3,4,5-Trimethoxybenzoyloxy)phenyl]methyl]-2-thioxo-4-imidazolidinone* (**6**-**25**). Yield 56%, mp: 194-196 °C. ^1^H-NMR (DMSO-*d*_6_): δ 3.01-3.04 (m, 2H, CH_2_), 3.77 (s, 3H, OCH_3_), 3.88 (s, 6H, OCH_3_), 4.59 (dd, *J* = 4.2, 5.1 Hz, 1H, CH), 7.17-7.27 (m, 4H, Ar-H), 7.41 (s, 2H, Ar-H), 10.12 (s, 1H, NH), 11.51 (s, 1H, NH); IR: 3432, 3275, 1845, 1747, 1592, 1519, 1335, 1204, 1092 cm^-1^. Anal calcd. for C_20_H_20_N_2_O_6_S: C, 57. 68, H, 4.84, N, 6.73; found: C, 57.36, H, 4.93, N, 6.67.

*5-[[4-(4-Phenylbenzoyloxy)phenyl]methyl]-2-thioxo-4-imidazolidinone* (**6**-**26**). Yield 58%, mp: 226-228 °C. ^1^H-NMR (DMSO-*d*_6_): δ 3.02-3.04 (m, 2H, CH_2_), 4.59 (dd, *J* = 4.2, 5.1 Hz, 1H, CH), 7.21-7.29 (m, 4H, Ar-H), 7.43-7.57 (m, 3H, Ar-H), 7.78-7.93 (m, 4H, Ar-H), 8.19-8.22 (m, 2H, Ar-H), 10.13 (s, 1H, NH), 11.53 (s, 1H, NH); IR: 3440, 3149, 1854, 1735, 1604, 1544, 1401, 1196, 1072 cm^-1^. Anal calcd. for C_23_H_18_N_2_O_3_S: C, 68. 64, H, 4.51, N, 6.96; found: C, 68.63, H, 4.44, N, 6.95.

*5-[[4-(4-tert-Butylbenzoyloxy)phenyl]methyl]-2-thioxo-4-imidazolidinone* (**6**-**27**). Yield 48%, mp: 237-239 °C. ^1^H-NMR (DMSO-*d*_6_): δ 3.01-3.03 (m, 2H, CH_2_), 4.57 (dd, *J* = 4.2, 5.1 Hz, 1H, CH), 7.19-7.27 (m, 4H, Ar-H), 7.61-7.64 (m, 2H, Ar-H), 8.04-8.07 (m, 2H, Ar-H), 10.12 (s, 1H, NH), 11.52 (s, 1H, NH); IR: 3440, 3159, 1852, 1741, 1607, 1547, 1402, 1197, 1073 cm^-1^. Anal calcd. for C_21_H_22_N_2_O_3_S: C, 65. 95, H, 5.80, N, 7.32; found: C, 65.81, H, 5.76, N, 7.29. ESI-MS m/z: 381 [M-H]^-^.

*5-[[4-(1-Naphthylacetoxy)phenyl]methyl]-2-thioxo-4-imidazolidinone* (**6**-**28**). Yield 51%, mp: 179-181 °C. ^1^H-NMR (DMSO-*d*_6_): δ 3.03-3.05 (m, 2H, CH_2_), 4.43 (s, 2H, CH_2_), 4.55 (dd, *J* = 4.2, 5.0 Hz, 1H, CH), 7.01-7.20 (m, 4H, Ar-H), 7.50-7.65 (m, 4H, Ar-H), 7.89-8.09 (m, 3H, Ar-H), 10.11 (s, 1H, NH), 11.51 (s, 1H, NH); IR: 3446, 3150, 1853, 1739, 1601, 1548, 1403, 1199, 1150, 1090 cm^-1^. Anal calcd. for C_22_H_18_N_2_O_3_S: C, 67. 67, H, 4.65, N, 7.17; found: C, 67.30, H, 4.73, N, 7.29. ESI-MS m/z: 389 [M-H]^-^.

*5-[[4-(3,5-Dinitrobenzoyloxy)phenyl]methyl]-2-thioxo-4-imidazolidinone* (**6**-**29**). Yield 41%, mp: 236-238 °C. ^1^H-NMR (DMSO-*d*_6_): δ 3.03-3.05 (m, 2H, CH_2_), 4.61 (dd, *J* = 4.2, 5.1 Hz, 1H, CH), 7.28-7.35 (m, 4H, Ar-H), 9.07-9.12 (m, 3H, Ar-H), 10.15 (s, 1H, NH), 11.55 (s, 1H, NH); IR: 3448, 3209, 1860, 1751, 1629, 1540, 1352, 1157, 1087 cm^-1^. Anal calcd. for C_17_H_12_N_4_O_7_S: C, 49. 04, H, 2.90, N, 13.46; found: C, 49.26, H, 3.21, N, 13.35.

*5-[[4-(2-Thenoyloxy)phenyl]methyl]-2-thioxo-4-imidazolidinone* (**6**-**30**). Yield 46%, mp: 208-210 °C. ^1^H-NMR (DMSO-*d*_6_): δ 3.02-3.05 (m, 2H, CH_2_), 4.60 (dd, *J* = 4.2, 5.1 Hz, 1H, CH), 7.18-7.24 (m, 4H, Ar-H), 7.26-7.33 (m, 1H, Thienyl-H), 8.01-8.11 (m, 2H, Thienyl-H), 10.11 (s, 1H, NH), 11.51 (s, 1H, NH); IR: 3445, 3209, 1781, 1733, 1612, 1546, 1408, 1211, 1056 cm^-1^. Anal calcd. for C_15_H_12_N_2_O_3_S_2_: C, 54. 20, H, 3.64, N, 8.43; found: C, 53.74, H, 3.45, N, 8.33.

*5-[[4-(Phenylacryloxy)phenyl]methyl]-2-thioxo-4-imidazolidinone* (**6**-**31**). Yield 40%, mp: 204-206 °C. ^1^H- NMR (DMSO-*d*_6_): δ 3.00-3.02 (m, 2H, CH_2_), 4.58 (dd, *J* = 4.2, 5.1 Hz, 1H, CH), 6.86 (d, *J* = 15.9 Hz, 1H, H-C=), 7.13 (d, *J* = 8.5 Hz, Ar-H), 7.24 (d, *J* = 8.5 Hz, Ar-H), 7.46-7.48 (m, 3H, Ar-H), 7.79-7.89 (m, 3H, Ar-H+H-C=), 10.17 (s, 1H, NH), 11.48 (s, 1H, NH); IR: 3440, 3150, 1780, 1739, 1630, 1548, 1502, 1185, 1156 cm^-1^. Anal calcd. for C_19_H_16_N_2_O_3_S: C, 64. 76, H, 4.58, N, 7.95; found: C, 64.64, H, 4.63, N, 8.03. ESI-MS m/z: 351 [M-H]^-^.

*5-[[4-(3,5-bis(Trifluoromethyl)phenylacetoxy)phenyl]methyl]-2-thioxo-4-imidazolidinone* (**6**-**32**). Yield 62%, mp: 161-163 °C. ^1^H-NMR (DMSO-*d*_6_): δ 2.49-2.52 (m, 2H, CH_2_), 4.27 (s, 2H, CH_2_), 4.56 (dd, *J* = 4.2, 5.1 Hz, 1H, CH), 7.09 (d, *J* = 8.6 Hz, 2H, Ar-H), 7.22 (d, *J* = 8.6 Hz, 2H, Ar-H), 8.04 (s, 1H, Ar-H), 8.16 (s, 2H, Ar-H), 10.08 (s, 1H, NH), 11.49 (s, 1H, NH); IR: 3442, 3168, 1782, 1747, 1546, 1502, 1391, 1290, 1086 cm^-1^. Anal calcd. for C_20_H_14_F_6_N_2_O_4_S: C, 50. 42, H, 2.96, N, 5.88; found: C, 50.80, H, 3.26, N, 6.01.

*5-[[4-(4-Fluorophenylacetoxy)phenyl]methyl]-2-thioxo-4-imidazolidinone* (**6**-**33**). Yield 52%, mp: 175-177 °C. ^1^H-NMR (DMSO-*d*_6_): δ 2.97-2.99 (m, 2H, CH_2_), 3.95 (s, 2H, CH_2_), 4.55 (dd, *J* = 4.2, 5.0 Hz,, 1H, CH), 7.03 (d, *J* = 8.5 Hz, 2H, Ar-H), 7.15-7.22 (m, 4H, Ar-H), 7.39-7.44 (m, 2H, Ar-H), 10.07 (s, 1H, NH), 11.48 (s, 1H, NH); IR: 3446, 3171, 1779, 1748, 1602, 1548, 1491, 1286, 1090 cm^-1^. Anal calcd. for C_18_H_15_FN_2_O_3_S: C, 60. 32, H, 4.22, N, 7.82; found: C, 60.46, H, 4.46, N, 8.08. ESI-MS m/z: 357 [M-H]^-^.

*5-[[4-(2-Furylformyloxy)phenyl]methyl]-2-thioxo-4-imidazolidinone* (**6**-**34**). Yield 74%, mp: 173-175 °C. ^1^H- NMR (DMSO-*d*_6_): δ 3.01-3.03 (m, 2H, CH_2_), 4.61 (dd, *J* = 4.2, 5.1 Hz, 1H, CH), 7.21-7.28 (m, 4H, Ar-H), 7.62-7.67 (m, 1H, Fu-H), 7.81-7.84 (m, 1H, Fu-H), 8.06-8.11 (m, 1H, Fu-H), 10.12 (s, 1H, NH), 11.51 (s, 1H, NH); IR: 3485, 3151, 1784, 1738, 1545, 1468, 1399, 1204, 1012 cm^-1^. Anal calcd. for C_15_H_12_N_2_O_4_S: C, 56. 96, H, 3.82, N, 8.86; found: C, 56.94, H, 4.07, N, 9.09.

#### 3.2.3. Synthesis of Compound **7**

To a stirred solution of 5-[(4-hydroxy phenyl)methyl]-2-thioxoimidazolidin-4-one (**5**, 2 mmol) and acetic anhydride (4 mmol) in dry actone (25 mL) and DMAP (0.02 g, 0.2 mmol) were added at room temperature. After about 10 h, the reaction is almost complete as monitored by TLC. After concentration under vacuum, the crude product was purified by column chromatography on silica gel (petroleum ether-EtOAc 3:1-2:1) to afford pale yellow solid compound **7**, yield 51%. Compound **7**, mp: 138-139 °C. IR: 3492, 3217, 3016, 2932, 1774, 1724, 1510, 1498, 1353, 1287, 1202, 1215, 1073, 1030 cm^-1^. ^1^H NMR (DMSO-*d*_6_): δ 12.45 (s, 1H), 7.05~6.98 (m, 4H), 4.93 (dd, *J* = 5.7, 2.7 Hz, 1H), 3.35 (dd, *J* = 14.1, 5.7 Hz, 1H), 3.11 (dd, *J* = 14.1, 2.7 Hz, 1H), 2.70 (s, 3H), 2.23 (s, 3H). Anal calcd for C_1__4_H_1__4_N_2_O_4_S: C, 54.95, H, 4.66, N, 9.03; found: C, 54.89, H, 4.61, N, 9.14.

#### 3.2.4. Synthesis of Compound **8**

To a stirred solution of 5-[(4-hydroxyphenyl)methyl]-2-thioxoimidazolidin-4-one (**5**, 2 mmol) in dry acetone (25 mL), benzoyl chloride (4 mmol) in 5 mL acetone and DMAP (0.02 g, 0.2 mmol) were added at 0 °C. Heated to reflux for 9 h after addition, the reaction is almost complete as monitored by TLC. After filtration and concentration under vacuum, the crude product was purified by column chromatography on silica gel (petroleum ether-EtOAc 4:1-2:1) to produce pale yellow solid compound **8**, yield 64%. Compound **8**, mp: 173-174. IR: 3458, 3067, 2893, 2773, 1764, 1651, 1595, 1499, 1349, 1214, 1067, 1012 cm^-1^. ^1^H NMR (DMSO-*d*_6_): δ 12.64 (s, 1H), 8.16~8.13 (m, 2H), 7.78~7.73 (m, 1H), 7.64~7.51 (m, 3H), 7.41~7.29 (m, 4H), 7.24~7.13 (m, 4H), 5.34 (dd, *J* = 5.7, 3.0 Hz, 1H), 3.41~3.34 (m, 2H). Anal calcd for C_24_H_1__8_N_2_O_4_S: C, 66.99, H, 4.28, N, 6.53; found: C, 66.96, H, 4.21, N, 6.51.

### 3.3. Bioassay of Herbicidal Activities

Herbicidal activity tests of compounds **6** were carried out in an illumination incubator. Ten seeds of *Zea mays* and five seeds of *Triticum aestivum* were chosen for the tests. Seedlings were grown in a plate containing a piece of filter paper and 2 mL solution of the tested compound (200 mg/L). *Arabidopsis thaliana* seeding were grown in 24-well sterile microliter plates, each well contained five sterilized *Arabidopsis thaliana* seedings and 100 μL solution of the tested compound (200 mg/L). Distilled water and metazachlor were used as the blank and positive control. The herbicidal activity was assessed as the inhibitory ratio in comparison with distilled water in the range from 0 to 100%. The tests were run triplicate for each compounds, the results were averaged and given in Table 1. The greenhouse test were performed according to pre-emergence and post-emergence treatment, *Echinochloa crus-galli*, *Lolium multiflorum, Poa annua, Setaria viridis, Abutilon theophrasti, Amaranthus retoflexus, Matricaria chamomilla, Stellaria media* were used as the target weeds.
